# Oropouche virus presenting in Italy after travel to Cuba

**DOI:** 10.1016/j.nmni.2024.101450

**Published:** 2024-07-02

**Authors:** Francesco Branda, Massimo Ciccozzi, Fabio Scarpa

**Affiliations:** Unit of Medical Statistics and Molecular Epidemiology, Università Campus Bio-Medico di Roma, Rome, Italy; Department of Biomedical Sciences, University of Sassari, Sassari, Italy

**Keywords:** Oropouche virus, Italy, Arbovirus, Epidemiology, Public health, Surveillance, Orthobunyavirus, Vector-borne diseases

Dear Editor,

Recently, the first European case of Oropouche fever was reported in Italy by the Department of Tropical Infectious Diseases and Microbiology of the IRCCS Sacro Cuore Don Calabria in Negrar, in a 26-year-old woman with no relevant medical history who has a recent history of travel to Cuba (https://tinyurl.com/oropouche-italy). Oropouche virus disease is an arboviral disease caused by the Oropouche virus (OROV) [1] belonging to the genus *Orthobunyavirus*, family *Bunyaviridae*. It is characterized by a spherical-shaped virion with a diameter ranging from 80 to 120 nm and its genome is tripartite and consists of three single-stranded RNA segments of negative polarity, named: (i) S (small), encoding for nucleoprotein N, which is essential for protection of the viral genome and regulation of RNA replication; (ii) M (medium), encoding for the envelope glycoproteins Gn and Gc, which are essential for virus entry into the host cell and induction of the immune response; L (large), encoding for RNA-dependent RNA polymerase, an enzyme critical for viral genome replication [1].

OROV can be transmitted to humans mainly through the bite of the mosquito *Culicoides paraensis*, which is present in forested areas and near water bodies, or some specimens of the mosquito *Culex quinquefasciatus*. Viral circulation includes both wild and epidemic cycles. In the wild cycle, primates, sloths, and possibly birds are the vertebrate hosts, although a definitive arthropod vector has not yet been identified. In the epidemic cycle, however, humans serve as the amplifying hosts, and OROV is transmitted predominantly through the bite of the mosquito *Culicoides paraensis*. The disease symptoms usually appear 3–8 days [2] after the bite of the vector insect and are largely similar to those of other tropical viral fevers such as dengue, Zika or chikungunya: high fever (above 39 °C) accompanied by headache, retroorbital pain, general malaise, myalgia, arthralgia, nausea, vomiting and photophobia. In about 60% of cases, after the first acute phase, symptoms recur in a less severe form, usually within two to ten days, but also after a month after the first appearance. To date, there is no evidence of human-to-human virus transmission, and there is no specific antiviral treatment or vaccine for Oropouche virus disease.

The virus was discovered in 1955 in the blood of a forestry worker from Trinidad and Tobago [3], and it is endemic in many South American countries, in both rural and urban communities. Outbreaks are periodically reported in Brazil, Bolivia, Colombia, Ecuador, French Guiana, Panama, Peru, and Trinidad and Tobago. In 2024, according to the latest data released by the Pan American Health Organization (PAHO), 5193 confirmed cases of Oropouche were reported in four countries in the Region of the Americas (https://www.paho.org/en/documents/epidemiological-alert-oropouche-region-americas-9-may-2024), of which 313 in Bolivia, 4583 in Brazil with the Amazon region accounting for 93% of cases, 38 in Bolivia with one additional case identified from Tabatinga, Brazil, and 259 in Peru, the highest number recorded in the country to date. 74 confirmed cases have also been identified in Cuba, such as reported by the Ministry of Public Health of Cuba on 27 May 2024, marking the first detection of the disease in the country (https://www.who.int/emergencies/disease-outbreak-news/item/2024-DON521). Consequently, the population is highly susceptible and there is a significant risk of detecting more cases.

Arboviruses such as dengue, Zika, chikungunya and Oropouche fever are increasingly becoming a public health emergency we must learn to live with. Experts point out that climate change and increased human travel threaten to make viruses once confined to the tropical belt endemic in our latitudes, such as OROV, which can occasionally be transmitted by secondary vectors such as *Culex quinquefasciatus* or *Aedes aegypti* mosquitoes, although it has not yet been reported in Italy.

Public health interventions, such as pest control and community awareness campaigns, remain vital to containing the risks associated to arboviruses. The integration of advanced diagnostic tools and continuous monitoring will enhance our ability to detect and respond to outbreaks swiftly, reducing the potential for widespread transmission. With the rapid advancement of sequencing technologies, a future is foreseeable in which we will have a more thorough understanding of the propagation and genetic recombination of OROV. In fact, the genetic variability among OROV genome segments and the geographic distribution of strains [3], [4] raise important questions about the virus's evolutionary dynamics. The scarcity of genetic data and the predominance of S-segment sequences limit our understanding. This lack of comprehensive information prevents a full view of virus evolution, overshadowing the dynamics of OROV in its reservoir hosts and vector populations. Understanding the genetic composition and variability of OROV is critical because the segmentation of its genome allows for reassortment events. These events may lead to the emergence of new variants with potential implications for disease severity, as observed in other *Bunyaviruses* such as Ngari virus [5].

Investing in the extensive and detailed collection of genetic data is therefore essential to better understand the biology of arboviruses and mitigate the risks associated with their infections. This will enable researchers to more effectively predict and prevent potential outbreaks. For example, in the specific case of Italy, the presence of the genomic sequence would have made it possible to determine whether the virus that infected the woman who traveled to Cuba was a typical Cuban lineage, a new variant, or a strain coming from other South American locations. In general, this type of information, if applied to all emerging cases, allows us to map and monitor the movements of different lineages, identify prevalence in different localities, and estimate the diffusion capacity. Furthermore, developing targeted interventions based on the genetic understanding can not only improve the outcomes of public health responses, but also guide the implementation of more effective strategies to contain the spread of viruses.

## Funding

None.

## CRediT authorship contribution statement

**Francesco Branda:** Conceptualization, Data curation, Investigation, Writing – original draft, Writing – review & editing. **Massimo Ciccozzi:** Conceptualization, Supervision, Validation, Writing – original draft, Writing – review & editing. **Fabio Scarpa:** Writing – original draft, Writing – review & editing.

## Declaration of competing interest

The authors declare that they have no known competing financial interests or personal relationships that could have appeared to influence the work reported in this paper.
